# Supplementary material for: Targeting Multiple Homeostasis-Maintaining Systems by Ionophore Nigericin Is a Novel Approach for Senolysis
Source: Int J Mol Sci. 2022 Nov 17;23(22):14251. doi: 10.3390/ijms232214251 (PMC9693507; doi:10.3390/ijms232214251)
Supplement: Supplementary file 1 [file ijms-23-14251-s001.zip › Supplementary Figure S1.pdf]

Targeting multiple homeostasis-maintaining systems by ionophore nigericin is a novel approach for senolysis

by Pavel I. Deryabin[1], Alla N. Shatrova[2], Aleksandra V. Borodkina[1,*]

[1]Mechanisms of cellular senescence group, Institute of Cytology of the Russian Academy of Sciences, Tikhoretsky Ave. 4, 194064, Saint-Petersburg, Russia
[2]Laboratory of intracellular membranes dynamic, Institute of Cytology of the Russian Academy of Sciences, Tikhoretsky Ave. 4, 194064, Saint-Petersburg, Russia
*Correspondence to Aleksandra V. Borodkina, Tikhoretsky ave. 4, 194064, St-Petersburg, Russia; borodkina618@gmail.com

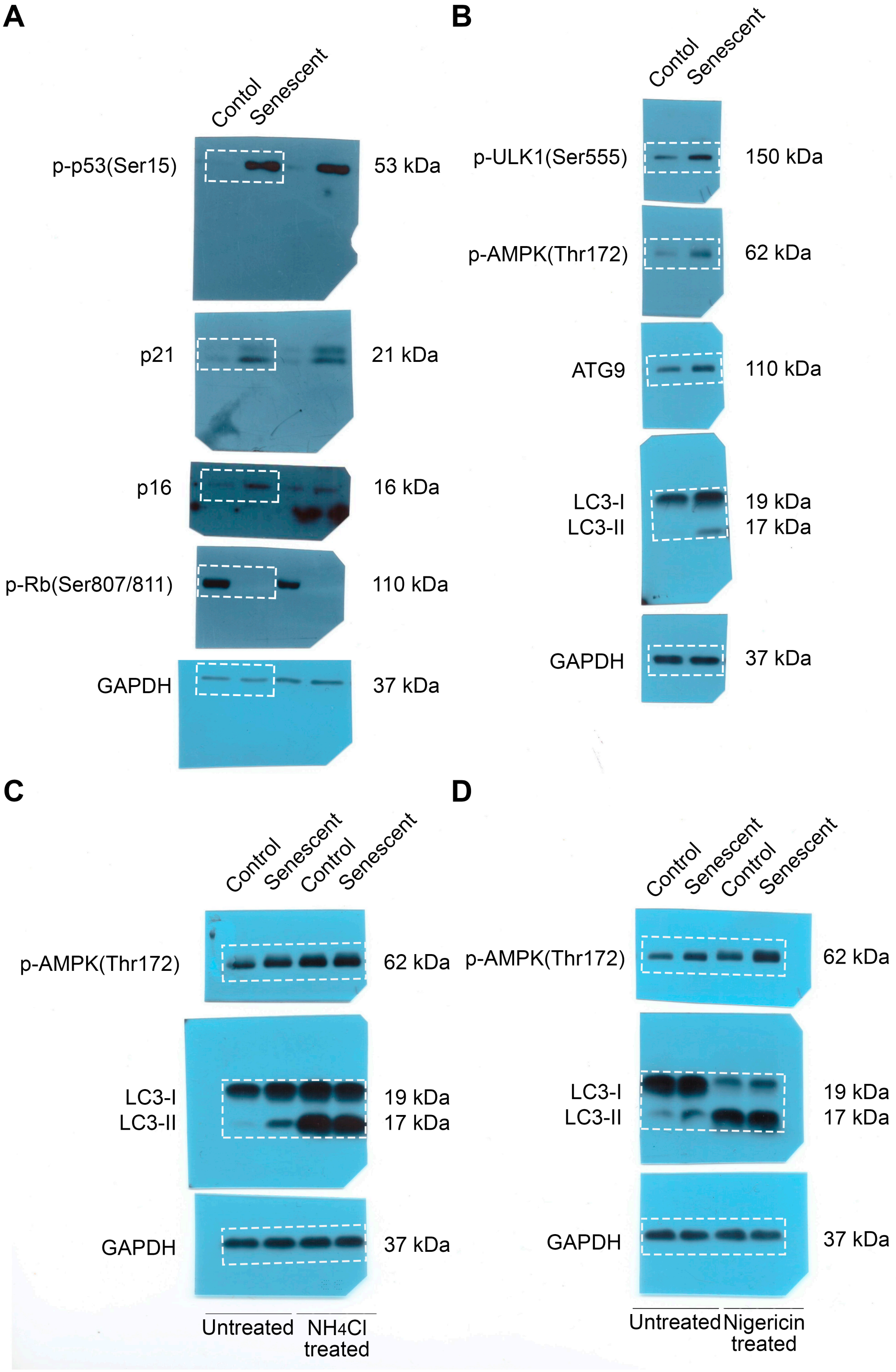

Supplementary Figure S1. Full-length blots presented in the article. (**A**) Blots for Figure 1B. (**B**) Blots for Figure 2H. (**C**) Blots for Figure 3H. (**D**) Blots for Figure 4E.
